# Phase 2b placebo-controlled trial of M72/AS01_E_ candidate vaccine to prevent active tuberculosis in adults 

**DOI:** 10.1056/NEJMoa1803484

**Published:** 2018-09-25

**Authors:** Olivier Van Der Meeren, Mark Hatherill, Videlis Nduba, Robert J Wilkinson, Monde Muyoyeta, Elana Van Brakel, Helen M Ayles, German Henostroza, Friedrich Thienemann, Thomas J. Scriba, Andreas Diacon, Gretta L. Blatner, Marie-Ange Demoitié, Michele Tameris, Mookho Malahleha, James C. Innes, Elizabeth Hellstrom, Neil Martinson, Tina Singh, Elaine Jacqueline Akite, Aisha Khatoon, Anne Bollaerts, Ann M. Ginsberg, Thomas G. Evans, Paul Gillard, Dereck R. Tait

**Affiliations:** GSK, Wavre and Rixensart, Belgium; South African Tuberculosis Vaccine Initiative (SATVI), Institute of Infectious Disease & Molecular Medicine and Division of Immunology, Department of Pathology, University of Cape Town, South Africa; Kenya Medical Research Institute (KEMRI/CRDR), Nairobi, Kenya; Wellcome Centre for Infectious Diseases Research in Africa, Institute of Infectious Disease and Molecular Medicine, University of Cape Town, South Africa, and Francis Crick Institute London, and Department of Medicine, Imperial College, London, United Kingdom; Centre for Infectious Disease Research in Zambia (CIDRZ), Lusaka, Zambiaa; TASK Applied Science, Cape Town, South Africa; Zambart, University of Zambia, Lusaka, Zambia, and London School of Hygiene and Tropical Medicine, London, United Kingdom; Centre for Infectious Disease Research in Zambia (CIDRZ), Lusaka, Zambia; Wellcome Centre for Infectious Diseases Research in Africa, Institute of Infectious Disease and Molecular Medicine, University of Cape Town, South Africa, and the Department of Internal Medicine, University Hospital of Zurich, Switzerland; South African Tuberculosis Vaccine Initiative (SATVI), Institute of Infectious Disease & Molecular Medicine and Division of Immunology, Department of Pathology, University of Cape Town, South Africa; TASK Applied Science, Cape Town, South Africa; Stellenbosch University, Cape Town, South Africa; AERAS, Rockville, United States of America; GSK, Wavre and Rixensart, Belgium; South African Tuberculosis Vaccine Initiative (SATVI), Institute of Infectious Disease & Molecular Medicine and Division of Immunology, Department of Pathology, University of Cape Town, South Africa; Setshaba Research Centre, Pretoria, Gauteng South Africa; The Aurum Institute, Klerksdorp and Tembisa Research Centres, South Africa; Be PART Yoluntu Centre, Paarl, South Africa; Perinatal HIV Research Unit (PHRU), Chris Hani Baragwanath Hospital, South African MRC Collaborating Centre for HIV/AIDS and TB, and NRF Centre of Excellence in Biomedical TB Research, University of the Witwatersrand Johannesburg, South Africa, and Johns Hopkins University Center for TB Research, Baltimore MD, United States of America; GSK, Wavre and Rixensart, Belgium; GSK, Wavre and Rixensart, Belgium; GSK, Wavre and Rixensart, Belgium; GSK, Wavre and Rixensart, Belgium; AERAS, Rockville, United States of America; AERAS, Rockville, United States of America; GSK, Wavre and Rixensart, Belgium; AERAS Global TB Vaccine Foundation, Cape Town, South Africa

## Abstract

**Background:**

A tuberculosis vaccine to interrupt transmission is urgently needed. We assessed the safety and efficacy of the candidate tuberculosis vaccine, M72/AS01_E_, against progression to bacteriologically-confirmed active pulmonary tuberculosis disease in adults with latent *Mycobacterium tuberculosis* (Mtb) infection.

**Methods:**

In a randomized, double-blind, placebo-controlled, phase 2b trial conducted in Kenya, South Africa and Zambia, human immunodeficiency virus (HIV)-negative adults aged 18-50 years with latent Mtb infection (positive by interferon-gamma release assay) were randomized (1:1) to receive two doses of either M72/AS01E or placebo intramuscularly on days 0 and 30. Clinical suspicion of tuberculosis was confirmed from sputum using a polymerase chain reaction test and/or mycobacterial culture.

**Results:**

This paper reports the primary analysis, conducted after a mean follow-up of 2.3 years. 1786 participants received M72/AS01_E_ and 1787 received placebo. In the vaccine group, 10 cases met the primary case definition (bacteriologically-confirmed active pulmonary tuberculosis confirmed prior to treatment, not associated with HIV infection) versus 22 cases in the placebo group (0.3 vs. 0.6 cases per 100 person-years, respectively): vaccine efficacy 54.0% (90% confidence interval 13.9-75.4; 95%CI 2.9-78.2; p=0.04). Solicited and unsolicited adverse events within 7 days post-injection were more frequent among M72/AS01_E_ recipients (91.2%) than placebo recipients (68.9%), the difference attributed mainly to injection site reactions and flu-like symptoms. Serious adverse events, potential immune-mediated diseases and deaths occurred with similar low frequencies between groups.

**Conclusions:**

M72/AS01_E_ was associated with a clinically acceptable safety profile and provided 54.0% protection for Mtb-infected adults against active pulmonary tuberculosis disease.

## Introduction

One-quarter of the global population is estimated to be infected with *Mycobacterium tuberculosis* (Mtb), and tuberculosis (TB) is the leading infectious cause of death worldwide.^1,2^ There were an estimated 10.4 million new TB cases and 1.7 million TB deaths in 2016. An effective TB vaccine for Mtb-infected persons would have a marked impact on TB control, including drug-resistant TB, through interruption of transmission.^3,4^ Modelling suggests the most effective contribution to TB control would be a vaccine preventing pulmonary TB in adolescents and young adults.^4^ The only licensed TB vaccine, BCG (bacille Calmette-Guérin), does not offer significant protection against pulmonary TB in Mtb-infected adults.^5^

The M72/AS01_E_ (GSK) candidate vaccine contains the M72 recombinant fusion protein derived from two immunogenic Mtb antigens (Mtb32A and Mtb39A), combined with the Adjuvant System AS01, which is also a component of the malaria (Mosquirix, GSK) and recombinant zoster (Shingrix, GSK) vaccines. The Mtb39A and Mtb32A components of the recombinant antigen elicited specific lymphoproliferation and/or interferon-gamma (IFN-γ) production in individuals with latent and active TB.^6-8^ In phase 2 studies, M72/AS01 exhibited a clinically acceptable safety profile, and induced humoral and cell-mediated immune (CMI) responses in healthy and human immunodeficiency virus (HIV)-infected individuals, Mtbinfected adults and adolescents, and in BCG-vaccinated infants (Table S1). 9-16

Despite the caveats associated with the available animal models,^17^ non-clinical evaluations (antigen-selection approach and *in vivo* preclinical data), clinical safety and immunogenicity evidence, based on the ability of the candidate vaccine to induce Th-1 type responses, supported a proof-of-concept human trial.^6-9,18-22^

We conducted a proof-of-concept phase 2b trial to evaluate M72/AS01_E_ in preventing bacteriologically-confirmed pulmonary TB in HIV-negative adults with Mtb infection, defined by a positive interferon-gamma release assay (IGRA) (www.clinicaltrials.gov. NCT01755598). This population was selected based on its higher incidence of pulmonary TB compared to IGRA-negative individuals, which allowed a smaller sample size for proofof- concept.^23^

## Methods

### Study design

The study is a multi-center, double-blind, randomized (1:1), placebo-controlled trial conducted in three TB endemic African countries (Kenya, South Africa and Zambia). The randomization was not stratified but was performed using a minimization algorithm accounting for gender and center (see Supplement for details). Eleven study sites were selected based on local TB prevalence and ability to perform the study according to Good Clinical Practice guidelines (GCP). The QuantiFERON TB Gold in-Tube assay (QFT, Qiagen) was used at the manufacturer’s recommended cut-off to identify latent Mtb infection. The study population is being followed up for 3 years after vaccination with M72/AS01_E_ or placebo. A pre-specified primary analysis was performed when all participants had completed at least 2 years of follow-up. Immunogenicity and reactogenicity were assessed in a subgroup of 300 participants. The final analysis after 3 years of follow-up and secondary study objectives including CMI responses will be reported in a subsequent publication.

The study was undertaken in accordance with GCP and the Declaration of Helsinki. The protocol was approved by ethics committees and regulatory authorities in each participating country. All participants provided informed consent. The study protocol is available at NEJM.org. Unblinded safety data are reviewed by an independent data monitoring committee (IDMC). Anonymized individual participant data and study documents can be requested for further research from www.clinicalstudydatarequest.com.

### Population

Adults 18-50 years of age were eligible if they were healthy or had stable chronic medical conditions, were HIV-negative, had no TB symptoms, were QFT-positive, and had a sputum sample negative for Mtb at baseline using polymerase chain reaction (PCR) (GeneXpert MTB/RIF, Cepheid). 

### Vaccination

Two doses of M72/AS01_E_ or placebo were administered intramuscularly into the deltoid one month apart. 

### Efficacy endpoints

The primary study objective was to evaluate M72/AS01_E_ efficacy to prevent active pulmonary TB according to the first case definition (primary endpoint; see Table 1 for case definitions). Secondary study objectives were vaccine efficacy (VE) according to additional case definitions, immunogenicity, safety and reactogenicity of the vaccine. 

**Table 1:** Case definitions of tuberculosis (TB)

Case definition	Clinical suspicion*	Culture results	PCR results	HIV status	Other condition
**1st definition (Primary endpoint):** Definite pulmonary TB disease not associated with HIV infection	X	Either or both positive		Negative	Sputum collected before initiation of TB treatment
Definition used for the sensitivity analysis of the primary endpoint: Definite pulmonary TB disease (any two positive sputum tests) not associated with HIV infection	X	Any two tests positive**		Negative	
2nd Definition: Definite PCR-positive pulmonary TB disease not associated with HIV infection	X	Any	Positive	Negative	
3rd Definition: Definite pulmonary TB, not associated with HIV-infection	X	Either or both positive		Negative	Sputum collected up to 4 weeks
4th Definition: Definite pulmonary TB	X	Either or both positive		Any	after initiation of TB treatment
5th Definition: Clinical TB (any location)	-	Any	Any	Any	Clinician has diagnosed TB disease and has decided to treat the patient
5th Modified definition: Clinical TB (any location) not associated with HIV-infection	-	Any	Any	Negative	

PCR = polymerase chain reaction

* Presenting with one or more of cough >1-2 weeks, fever >1 week, night sweats, weight loss, pleuritic chest pain, hemoptysis, fatigue, shortness of breath on exertion.

** either two positive cultures, or two positive by PCR, or one positive by culture and one by PCR.

Possible deaths due to TB have not been included in any of the case definitions unless the case definition criteria as stated were met.

### Evaluation of safety and reactogenicity

Serious adverse events (SAEs), potential immune-mediated diseases (pIMDs) and pregnancies were recorded until 6 months after the second vaccination. SAEs deemed related to study product were recorded until study end. Unsolicited AEs were recorded for 30 days after each dose. Local and systemic symptoms were solicited from the subgroup using diary cards for 7 days after each injection. Laboratory testing for clinical chemistry and hematology was performed in the subgroup on Days 0, 7, 30 and 37. 

### Evaluation of immunogenicity

Blood samples were collected from the subgroup before dose 1, one month post-dose 2, and annually until year 3. Anti-M72 IgG antibodies were measured using enzyme-linked immunosorbent assay (ELISA) as previously described (cut-off 2.8 ELISA units/ml).^13^

### TB surveillance

TB surveillance used both active (visits, phone calls and text messages), and passive (selfreporting) methods. Participants with clinical suspicion of pulmonary TB provided three sputum samples collected over one week for PCR and liquid culture by Mycobacterial Growth Indicator Tube. Samples were preferably to be taken before initiation of TB treatment, but samples collected up to 4 weeks after treatment initiation were accepted (Table 1, case definitions 3 and 4). Diagnostic and treatment decisions were made by treating non-study physicians. HIV retesting and screening for diabetes (HbA1c) were performed in all participants with confirmed TB disease. 

### Statistical analysis

Using a log rank test with 80% power assuming a true VE of 70% (hazard ratio of 30%) and a 2-sided 10% significance level, 21 cases were required for a fixed sample design assuming proportional hazard rates. To obtain 21 cases, assuming a mean yearly attack rate of 0.55% in the control group, 2 years of follow-up for each subject and an attrition rate of 15% over the 2-year period, 3,506 participants needed to be enrolled. As per protocol, the primary analysis could occur at 21 cases or at completion of 24-month follow-up.

VE was analyzed in the according-to-protocol efficacy cohort, using a Cox proportional hazard regression models (VE=1-hazard ratio) with 90% confidence intervals (CIs) and Wald p-value. Descriptive *post-hoc* 95% CIs are also provided. The primary endpoint was met if the lower limit of the 2-sided 90% CI for VE against bacteriologically-confirmed pulmonary TB (first case definition) was >0%. If the primary endpoint was met, the first secondary endpoint (VE for the second case definition) was to be analyzed using the same success criterion. A pre-planned exploratory analysis compared the effect of 6 pre-specified covariates (giving 14 subgroups) on VE (interpretation should be performed cautiously as the risk of having at least one false significant result ranges between 51%–77%).

The total vaccinated cohort (all subjects who received at least one vaccination) was used to assess safety. Analysis of immunogenicity was performed on the according-to-protocol immunogenicity cohort for the subgroup. 

Statistical analyses were performed with SAS version 9.2 or above on SAS Drug Development. Only the external statisticians and IDMC have been unblinded at the level of individual subject data. 

The Supplementary Appendix provides the eligibility criteria and screening procedures, vaccine and placebo composition, safety monitoring and surveillance activities.

## Reults

Out of 3,575 randomized participants, 3,573 received at least one dose of M72/AS01E or placebo between August 2014 and November 2015. The mean age of participants was 28.9 (standard deviation [SD] 8.3 years); 43% were women. The study groups were balanced in terms of pre-specified demographic characteristics (Table S2). 

### Vaccine efficacy

**Figure 1:**
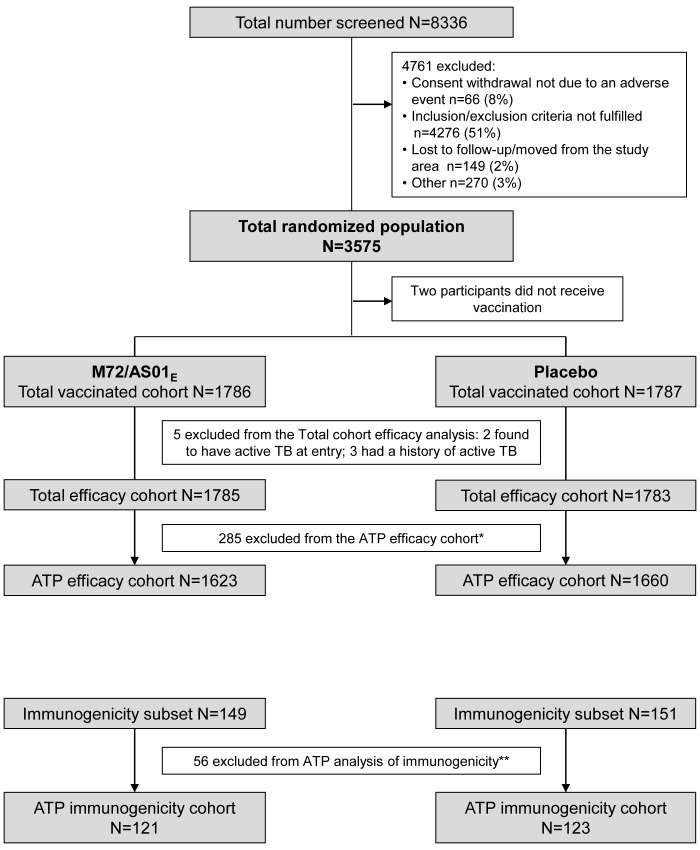
Study flow ATP = according to protocol, N = number of participants, TB = tuberculosis *Participants eliminated from the ATP efficacy cohort: administration of vaccine forbidden in the protocol (19), randomization error (2), randomization code broken at the investigator site (1), study vaccine not administered according to protocol (3), did not receive two vaccine doses (236), did not enter the efficacy evaluation period one month post-dose 2 (11), active tuberculosis (any case definition) diagnosed up to one month post-dose 2 (1), administration of medication forbidden by the protocol (2), non-compliance with vaccination schedule (3), did not meet inclusion/exclusion criteria (7). **Participants eliminated from the ATP immunogenicity cohort: administration of vaccine forbidden in the protocol (4), sputum Mtb positive at baseline (1), did not meet inclusion/exclusion criteria (1), concomitant infection (active TB) related to the vaccine which may influence immune response (1), concomitant infection (became HIV -infected) not related to the vaccine which may influence immune response (7), non- compliance with the vaccination schedule (3), non-compliance with the blood sampling schedule (9), essential serological data missing (all post-vaccination time points month 2 and month 12 missing) (15), did not receive two vaccine doses (15).

**Figure 2:**
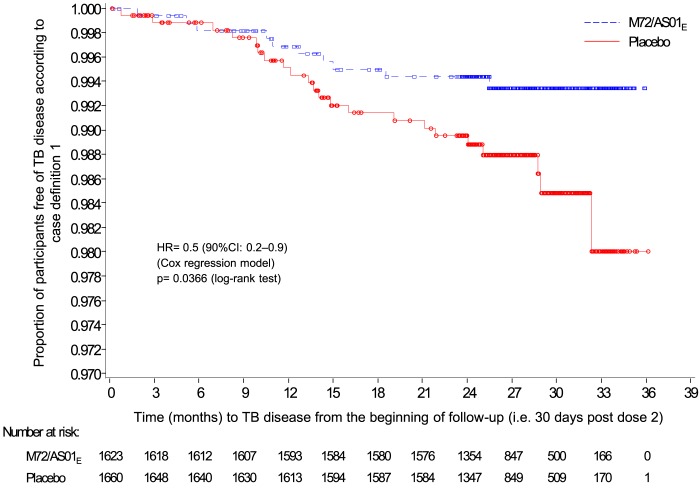
Kaplan- Meier estimate of definite pulmonary tuberculosis disease not associated with HIV -infection (first case definit ion) (According to protocol efficacy cohort) The decreased number at risk after 24 months reflects the participants for whom follow-up after this time point had not occurred at the data lock point. HR = hazard ratio CI = confidence interval TB = tuberculosis

There were 3,283 participants included in the according-to-protocol efficacy analysis (Figure 1). Ten cases of active pulmonary TB in the vaccine group and 22 cases in the placebo group met the primary case definition after mean follow-up of 2.3 years (SD 0.4) (Table 2). The incidence of pulmonary TB (first case definition) per 100 person-years was 0.3 in the M72/AS01_E_ and 0.6 in the placebo group, with overall VE 54.0% (90% CI 13.9-75.4; 95% CI 2.9-78.2; p=0.04). Analysis using a Cox regression model adjusted for country, gender, diabetes, age strata, smoking, and BCG history, gave nearly identical results (data not shown). VE for the second case definition (secondary endpoint) was 58.3% (90% CI 12.8- 80.1; 95% CI -0.5-82.7; p=0.05), and ranged from 28%–36% for protocol-defined case definitions 3 to 5 (Table 2). Kaplan-Meier curves are displayed in Figure 2 for the first case definition. Results were comparable in the total vaccinated efficacy cohort analysis (VE 57.0% [90% CI: 19.9-76.9; 95% CI: 9.7-79.5]; Table 2). 

**Table 2:** Vaccine efficacy of M72/AS01_E_ versus placebo against pulmonary tuberculosis (TB) in adults with evidence of TB infection (unadjusted Cox regression model) N = number of participants included in each group, n = number of participants having pulmonary TB according to the definitions specified in Table 1, person-years = sum of follow-up periods (expressed in years). Follow-up starts 30 days after dose 2 for according-to-protocol analysis and from the day of dose 1 for total vaccinated efficacy cohort analysis, and ends for both analyses at the first occurrence of pulmonary TB for cases, or for non-cases at either the individual end of the follow-up or at the data lock point, whichever comes first. CI = confidence interval, P-value = 2-sided p-value from Cox regression model

According-to-protocol efficacy cohort	M72/AS01_E_ N=1623			Placebo N=1660			Vaccine efficacy		
TB case definition	n	Person-years	Rate per 100 person-years (90% CI)	n	Person-years	Rate per 100 person-years (90% CI)	% (90% CI)	% (95% CI)	p-value
1st definition	10	3707.03	0.3 (0.2-0.5)	22	3747.43	0.6 (0.4-0.8)	54.0 (13.9-75.4)	54.0 (2.9-78.2)	0.04
Sensitivity analysis	5	3709.42	0.1 (0.1-0.3)	17	3751.23	0.5 (0.3-0.7)	70.3 (31.3-87.1)	70.3 (19.4-89.0)	
2nd definition	7	3709.42	0.2 (0.1-0.4)	17	3751.23	0.5 (0.3-0.7)	58.3 (12.8-80.1)	58.3 (-0.5-82.7)	0.05
3rd definition	16	3707.03	0.4 (0.3-0.7)	25	3747.43	0.7 (0.5-0.9)	35.3 (-9.5-61.8)	35.3 (-21.2-65.5)	
4th definition	17	3707.03	0.5 (0.3-0.7)	27	3747.43	0.7 (0.5-1.0)	36.4 (-5.9-61.8)	36.4 (-16.8-65.3)	
5th definition	21	3711.87	0.6 (0.4-0.8)	30	3753.43	0.8 (0.6-1.1)	29.2 (-13.1-55.7)	29.2 (-23.7-59.5)	
5th modified definition	20	3711.87	0.5 (0.4-0.8)	28	3753.03	0.7 (0.5-1.0)	27.7 (-17.0-55.3)	27.7 (-28.3-59.3)	

Total vaccinated efficacy cohort	M72/AS01_E_ N=1785			Placebo N=1783			Vaccine efficacy		
TB case definition	n	Person-years	Rate per 100 person-years (90% CI)	n	Person-years	Rate per 100 personyears (90% CI)	% (90% CI)	% (95% CI)	p-value
1st definition	10	4301.70	0.2 (0.1-0.4)	23	4253.72	0.5 (0.4-0.8)	57.0 (19.9-76.9)	57.0 (9.7-79.5)	0.03
Sensitivity analysis	5	4304.09	0.1 (0.1-0.2)	17	4258.75	0.4 (0.3-0.6)	70.9 (32.9-87.4)	70.9 (21.2-89.3)	
2nd definition	7	4304.09	0.2 (0.1-0.3)	17	4258.75	0.4 (0.3-0.6)	59.3 (14.8-80.5)	59.3 (1.8-83.1)	<0.05
3rd definition	17	4301.70	0.4 (0.3-0.6)	26	4253.72	0.6 (0.4-0.8)	35.4 (-7.9-61.3)	35.4 (-19.1-64.9)	
4th definition	18	4301.70	0.4 (0.3-0.6)	28	4253.72	0.7 (0.5-0.9)	36.5 (-4.4-61.3)	36.5 (-14.9-64.9)	
5th definition	23	4306.54	0.5 (0.4-0.8)	30	4260.96	0.7 (0.5-1.0)	24.1 (-19.7-51.9)	24.1 (-30.6-55.9)	
5th modified definition	22	4306.54	0.5 (0.4-0.7)	28	4260.55	0.7 (0.5-0.9)	22.2 (-24.2-51.3)	22.2 (-35.9-55.5)	

A planned sensitivity analysis of the first case definition was retricted to participants positive for Mtb by at least two diagnostic tests (culture and/or PCR) performed on the sputa collected (Table S3). This analysis included 5 cases in the M72/AS01E group and 17 cases in the placebo group; VE was 70.3% (90% CI 31.3-87.1%; p=0.02) (Table 2). Piece-wise analysis of cases (first case definition) occurring before versus after the median follow-up time (1.12 years) showed VE of 39.0% (90% CI -42.5-73.9) in the first period and 66.5% (90% CI 13.3-87.0) in the second period. 

Pre-specified subgroup analyses using case definition 1 showed VE in men of 75.2% (p=0.03) and in women of 27.4% (p=0.52), and VE in participants aged <=25 years of 84.4% (p=0.01) and VE for those aged >25-50 years of 10.2% (p=0.82) (Table 3). A post-hoc hierarchical test was performed to assess the interaction between group and gender (p=0.31) and between group and age (p=0.07) in the complete model containing all main effects as well as the two interaction terms (Table S4). 

**Table 3:** Vaccine efficacy of definite pulmonary tuberculosis disease not associated with HIV-infection (case definition 1) for each covariate and overall (unadjusted Cox regression model, according-to-protocol efficacy cohort)

Covariates	Group	N	n	Person-years	Rate per 100 person-years (90% CI)	Vaccine efficacy	
						% (90% CI)	% (95% CI)
Overall	M72/AS01_E_	1623	10	3707.03	0.3 (0.2-0.5)	54.0 (13.9-75.4)	54.0 (2.9-78.2)
	Placebo	1660	22	3747.43	0.6 (0.4-0.8)		
Diabetes (No)	M72/AS01_E_	1615	10	3688.14	0.3 (0.2-0.5)	53.9 (13.8-75.4)	53.9 (2.8-78.2)
	Placebo	1655	22	3735.22	0.6 (0.4-0.8)		
Diabetes (Yes)	M72/AS01_E_	7	0	16.29	0.0	0.0	0.0
	Placebo	5	0	12.21	0.0		
Female	M72/AS01_E_	679	7	1572.39	0.4 (0.2-0.8)	27.4 (-63.4-67.7)	27.4 (-90.8-72.4)
	Placebo	708	10	1627.29	0.6 (0.4-1.0)		
Male	M72/AS01_E_	944	3	2134.63	0.1 (0.1-0.4)	75.2 (28.3-91.4)	75.2 (12.2-93.0)
	Placebo	952	12	2120.13	0.6 (0.4-0.9)		
Kenya	M72/AS01_E_	242	2	549.09	0.4 (0.1-1.2)	-101.6 (-1411.7-73.1)	-101.6 (-2123.7-81.7)
	Placebo	246	1	550.84	0.2 (0.0-0.9)		
South Africa	M72/AS01_E_	1307	8	3008.71	0.3 (0.1-0.5)	59.3 (19.0-79.6)	59.3 (7.6-82.1)
	Placebo	1344	20	3058.97	0.7 (0.5-0.9)		
Zambia	M72/AS01_E_	74	0	-	-		
	Placebo	70	1	-	-		
Current smoker	M72/AS01_E_	831	7	1891.62	0.4 (0.2-0.7)	53.3 (0.8-78.0)	53.3 (-14.6- 80.9)
	Placebo	842	15	1891.36	0.8 (0.5-1.2)		
Not a current smoker	M72/AS01_E_	791	3	1812.82	0.2 (0.1-0.4)	56.0 (-36.9-85.9)	56.0 (-70.1- 88.6)
	Placebo	818	7	1856.06	0.4 (0.2-0.7)		
Age ≤25 years	M72/AS01_E_	705	2	1599.77	0.1 (0.0-0.4)	84.4 (45.7-95.5)	84.4 (31.0-96.5)
	Placebo	724	13	1616.66	0.8 (0.5-1.3)		
Age >25 years	M72/AS01_E_	918	8	2107.25	0.4 (0.2-0.7)	10.2 (-99.6-59.6)	10.2 (-132.7-65.4)
	Placebo	936	9	2130.77	0.4 (0.2-0.7)		
No BCG history or scar	M72/AS01_E_	136	*1*	-	-		
	Placebo	149	*1*	-	-		
BCG history and/or scar	M72/AS01_E_	1243	8	2823.92	0.3 (0.2-0.5)	55.8 (11.0-78.0)	55.8 (-1.8-80.8)
	Placebo	1247	18	2808.34	0.6 (0.4-0.9)		
Unknown BCG status	M72/AS01_E_	243	1	555.68	0.2 (0.0-0.9)	73.1 (-69.1-95.7)	73.1 (-140.5-97.0)
	Placebo	246	4	591.74	0.7 (0.3-1.5)		

N = number of participants included in each group, n = number of participants meeting case definition 1, BCG = bacille Calmette-Guérin, person-years = sum of follow-up period (up to the first occurrence of pulmonary TB, or to either the individual end of the follow-up or to the data lock point, which ever occurred first) expressed in years, CI = confidence interval, p-value = Two-sided p-value from Cox regression model, *1* = one case that remains blinded

### Reactogenicity and safety

The percentage of participants who experienced at least one SAE within 6 months postvaccination was similar between the groups (1.6% in the M72/AS01_E_ group and 1.8% in the placebo group) (Table 4). One SAE in each group was considered causally related to vaccination (pyrexia and hypertensive encephalopathy, currently blinded to group). pIMDs were reported by two participants in the M72/AS01_E_ group and 5 in the placebo group. There were 24 deaths (14 trauma-related) during the study, 7 in the M72/AS01_E_ group (5 trauma-related) and 17 in the placebo group (9 trauma-related) (Table 4). No death was assessed as related to study vaccination. One participant died of pneumonia for whom there was also a suspicion of intestinal TB, but this latter diagnosis was not confirmed. Vaccination did not significantly affect hematology and biochemistry parameters (Figure S1). A *post-hoc* analysis showed 33 pregnancies, of which 28 resulted in delivery of a healthy infant. There were three ectopic pregnancies, one spontaneous abortion, and one pregnant woman was lost-to-follow-up. No birth defects were noted. Regular IDMC review of unblinded safety data resulted in recommendations to continue the study unchanged. 

**Table 4:** Vaccine safety summary (Total vaccinated cohort)

	M72/AS01_E_		Placebo		
	N=1786		N=1787		
	n	% (95% CI)	n	% (95% CI)	RR (95% CI)
*30 days post-vaccination*					
At least one unsolicited symptom	1203	67.4 (65.1-69.5)	812	45.4 (43.1-47.8)	1.48 (1.35-1.62)
At least one causally-related unsolicited symptom	992	55.5 (53.2-57.9)	371	20.8 (18.9-22.7	2.68 (2.37-3.02)
At least one grade 3 symptom	234	13.1 (11.6-14.8)	124	6.9 (5.8-8.2)	1.89 (1.51-2.37)
At least one causally-related grade 3 symptom	177	9.9 (8.6-11.4)	27	1.5 (1.0-2.2)	6.56 (4.36-10.23)
At least one SAE	10	0.6 (0.3-1.0)	17	1.0 (0.6-1.5)	
At least one causally-related SAE	1	0.1 (0.0-0.3)	1	0.1 (0.0-0.3)	
*Within 6 months post-vaccination*					
At least one SAE	29	1.6 (1.1-2.3)	33	1.8 (1.3-2.6)	
At least one causally-related SAE	1	0.1 (0.0-0.3)	1	0.1 (0.0-0.3)	
pIMD	2	0.1 (0.0-0.4)	5	0.3 (0.1-0.7)	
Immune thrombocytopenic purpura	1 case that remains blinded				
Basedow’s (Graves) disease	1 case that remains blinded				
Gout	1 case that remains blinded				
Optic neuritis	2 cases that remain blinded				
Erythema multiforme	1 case that remains blinded				
Rash morbilliform	1 case that remains blinded				
*Whole study period*					
Fatal SAE, all	7	0.4 (0.2-0.8)	17	1.0 (0.6-1.5)	
Injury (gunshot, stab wound, road traffic accident, burn)	5	-	8	-	
Cardiac disorder	1 case that remains blinded				
Unknown cause/ Sudden death	3 cases that remain blinded				
Hepatic cirrhosis and hepatic encephalopathy	1 case that remains blinded				
Acute HIV infection	1 case that remains blinded				
Pneumonia and gastrointestinal tuberculosis suspicion	1 case that remains blinded				
Cerebrovascular accident	1 case that remains blinded				
Completed suicide	1 case that remains blinded				
Dyspnoea (drug overdose)	1 case that remains blinded				
Not coded (stab wound)	1 case that remains blinded				
Fatal SAE, causally-related	0	-	0	-	

pIMD = potential immune-mediated disease, SAE = serious adverse event, CI = confidence interval, N = number of participants in the indicated cohort, n = number of participants reporting the symptom; RR = relative risk.

More participants reported unsolicited AE in the M72/AS01_E_ group (67.4%) than in the placebo group (45.4%). The excess was driven by injection-site reactions and influenza-like symptoms (Table S5). Swelling reactions larger than 100mm diameter were reported by 53 participants (3.0%) in the M72/AS01_E_ group and by one participant in the placebo group. The median duration of these large swelling reactions was 4 days. 

In the subgroup, local and systemic solicited symptoms were reported more frequently by M72/AS01_E_ recipients than placebo recipients (Table S6). Among local solicited symptoms, pain was the most frequently reported (81.8% of M72/AS01_E_ recipients and 34.4% of placebo recipients, with 24.3% and 3.3%, respectively, reporting grade 3 pain). Redness and swelling were uncommon in both groups. Fatigue, headache, malaise, or myalgia was each reported by 58.1%-68.9% of M72/AS01_E_ recipients and 26.5%-47.0% of placebo recipients. Fever >38.0°C was reported by 18.9% and 6.6%, respectively. Fever >39.5°C was uncommon (4.1% versus 1.3%, respectively). 

The immunogenicity results indicate 100% of participants in the M72/AS01E group seroconverted at Month 2 and 99% were still seropositive at Month 12 (Figure S2). Figure S3 elaborates on the clinical relevance of this study that could be shared with patients by healthcare professionals. 

## Discussion

There is no TB vaccine recommended for use in Mtb-infected adults, who represent a large reservoir of future cases of active TB. Here, we demonstrate that protection against TB disease may be achieved by vaccination of Mtb-infected adults with an adjuvanted subunit vaccine containing two Mtb proteins. The finding of efficacy for the primary endpoint was supported by the more stringent sensitivity analysis, and by the analysis of the second case definition. Less stringent case definitions 3-5 showed similar, but non-significant, trends for efficacy. This is the first efficacy trial with M72/AS01_E_, and the results confirm the clinically acceptable safety and reactogenicity profile observed previously. Antibody responses were in the same range as previously observed in M72/AS01_E_-vaccinated adults living in TB endemic regions.^9,10^

Since the trial included only Mtb-infected individuals, it is not possible to determine the extent to which Mtb infection influences VE. In previous TB efficacy trials, the viral-vectored candidate vaccine MVA85A showed no additional protection beyond that provided by BCG in Mtb-uninfected infants;^24^ multiple doses of inactivated *M. vaccae (obuense)* administered to HIV-infected adults reduced the hazard of definite TB, which was a secondary endpoint, by 39%, with no effect modification by baseline Mtb infection status.^25^ A comprehensive global TB vaccination strategy should be targeted at both Mtb-uninfected and -infected adolescents and adults.^4^ Our study in Mtb-infected adults complements the findings of a recent trial that demonstrated 45% efficacy of BCG revaccination for protection of Mtb non-infected adolescents against sustained QFT seroconversion (*In press*).^26^ These results suggest a potential role for M72/AS01_E_ among vaccination strategies against TB. 

Recent research suggests that progression from latent Mtb infection to active TB is not a single definitive event, but rather a transition through a spectrum of inflammatory and infected states reflecting the activity of individual granulomas.^27^ Clinically, this spectrum results in heterogeneous disease states within and between individuals. In this study, participants with clinical suspicion of TB underwent diagnostic investigation. Approximately one-third of confirmed pulmonary TB cases were only confirmed by a single test out of the six performed (either culture or PCR). ‘Single positive’ cases were evenly distributed between the vaccine and placebo arms and became positive by culture (7 cases) after an unusually long period or by PCR (3 cases) after an unusually high number of amplification cycles. We hypothesize that active surveillance of study participants detected pulmonary TB with low bacterial load, consistent with early stages of disease or reinfection. Interestingly, three (out of 10) ‘single positive’ participants (blinded as to group) did not receive TB treatment and remain well, suggesting successful immune control and lack of disease progression. The sensitivity analysis suggested higher VE in participants with at least two positive tests, consistent with higher bacterial load. Piece-wise and time-to-event analyses did not demonstrate significant VE during year 1. We hypothesize this may be because at least some individuals who developed active TB during this time already had incipient TB at baseline, against which the vaccine could not be expected to have impact, or that the study did not have power to demonstrate a difference in the first year, or that this was a chance finding. Whilst we made reasonable efforts to exclude participants with active TB at screening (single PCR test on one sputum specimen), a limitation of the study was that we could not exclude that early active cases were missed, given the frequently low bacillary load and sporadic nature of bacillary shedding in early stages of TB disease.^28^

Unexpectedly, we observed a higher point estimate for VE in men than women (attack rate in the placebo group 0.6 per 100 person-years for men and women), and in those aged <=25 years versus 26-50 years (attack rate 0.8 versus 0.4 per 100 person-years, respectively). A *post-hoc* demographic analysis showed an imbalance in gender in the group aged <=25 years (66% men and 34% women), while the older age group was well-balanced, suggesting that the apparent difference observed by gender was confounded by the effect of age and is artefactual. Additionally, in a *post-hoc* interaction test, VE did not seem to be different by gender (p=0.3), while efficacy tended to be heterogeneous across age groups (p=0.07 in a hierarchical model containing both interactions). Interpretation of all *post-hoc* and exploratory subgroup analyses should be performed cautiously because the study was not powered to detect differences between subgroups and multiplicity was not accounted for. 

Age could potentially affect VE through a differential vaccine effect according to time since primary Mtb infection or BCG priming.^29^ We hypothesize that those further from primary infection are more likely to have infection under immune system control, with little additional benefit conveyed by vaccination. Increasing age is associated with increased probability of more remote infection based on several studies 30-34and screening data from the current study, in which 55.1%–66.6% of the screened individuals were already Mtb-infected.^31^ Alternatively, the circumstances that lead to reactivation may be less amenable to immunologic control by booster vaccination further from primary BCG vaccination or initial Mtb infection, and therefore the benefits of vaccination may be more limited. Given the age of the study population, immune senescence is unlikely to impact VE. 

PCR had sensitivity of 80% compared to culture (Table S7), consistent with more events meeting the first case definition than the second. Future VE trials should therefore utilize automated liquid culture in addition to PCR to maximize case detection. TB treatment of adult drug-sensitive pulmonary TB leads to negative sputum culture and PCR in 8 weeks in some participants;^35^ therefore case definitions 3 and 4 likely underestimate TB incidence.

 Strengths of the study were the inclusion of a large, well-defined cohort, exclusion of active TB disease at baseline, statistical power to address the primary endpoint, and the use of alternative case definitions for the efficacy endpoint that reflect applicability in the real world. Finally, 99% of participants consented to biobanking of pre- and post-vaccination blood samples. These samples offer the opportunity to discover potential immune correlates of vaccine-mediated protection against TB, which if confirmed, will be critical to reduce the size of future efficacy trials (www.clinicaltrials.gov. NCT02097095). 

In conclusion, M72/AS01_E_ had an acceptable safety and reactogenicity profile, and significantly reduced the incidence of pulmonary TB in healthy Mtb-infected HIV-negative adults. These promising results provide a unique opportunity to better understand the mechanisms by which this vaccine confers protection against TB and could lead to future improvements in global tuberculosis control. 

## Supplementary Appendix

Supplementary Material
