# Supplementary material for: Phase 2b placebo-controlled trial of M72/AS01E candidate vaccine to prevent active tuberculosis in adults
Source: N Engl J Med. Author manuscript; Available in PMC 2018 Sep 25. (PMC6151253; doi:10.1056/NEJMoa1803484)
Supplement: Supplementary Material [file 1803484_VanDerMeeren_Supplement.pdf]

Phase 2b placebo-controlled trial of M72/AS01E candidate vaccine to prevent active tuberculosis in adults

Supplementary Appendix

List of Principal investigators 2
Acknowledgements 3
Supplementary methods 6
- Inclusion and exclusion criteria 6
- Screening procedures 8
- QuantiFERON TB Gold In-Tube assay 8
- Composition of vaccine and placebo 9
- Treatment allocation and randomization 9
- Safety monitoring plan 9
- Surveillance for pulmonary TB 12
- PCR (Xpert MTB/RIF) for Mtb detection 13
- Statistical analysis of vaccine efficacy 13

Supplementary references 28

List of Supplementary Tables and Figures

Table S 1 Summary of available clinical data using Mtb72F or M72 15

Table S 2 Demographic and baseline characteristics of participants (Total vaccinated cohort) 17

Table S 3 Number of tests (PCR and/or culture) positive for each case under case definition 1 (According to protocol cohort for efficacy – post-hoc analysis) 18

Table S 4 P-values in the Cox regression model with group, gender, age, gender by group interaction and age by group interaction for case definition 1 (ATP cohort for efficacy) 19

Table S 5 Unsolicited events; all events and those reported by at least 1% of participants within 30 days after each dose (Total vaccinated cohort) 20

Table S 6 Local and general solicited symptoms, all and grade 3, occurring until day 7 post-vaccination (sub-cohort, Total vaccinated cohort) 21

Table S 7 Concordance of PCR and microbiological culture tests for sputum samples from all suspected pulmonary tuberculosis cases (Total vaccinated cohort for Efficacy) 23

Figure S 1 Percentage of participants outside the normal ranges and at the different grades of severity for hematology, biochemistry (Total vaccinated cohort in the immunogenicity subset, grading 1-4 according to FDA standards) 24

Figure S 2 Immunogenicity before and one month and 12 months post-dose 2 (ATP immunogenicity sub-cohort) 26

Figure S 3: “Focus on the Patient” section 27

List of Principal investigators

Kenya

- Kenya Medical Research Institute: Videlis Nduba

South Africa

- The Aurum Institute (Klerksdorp and Tembisa Sites): James C. Innes
- Be Part Yoluntu Centre: Elizabeth Hellstrom
- Wellcome Centre for Infectious Diseases Research in Africa: Robert J. Wilkinson
- Perinatal HIV Research Unit: Neil Martinson
- South African Tuberculosis Vaccine Initiative: Mark Hatherill
- Setshaba Research Centre: Mookho Malahleha
- Task Applied Science: Andreas Diacon

Zambia

- Centre for Infectious Diseases Research in Zambia: Monde Muyoyeta, German Henostroza
- Zambart: Helen M Ayles

Acknowledgements

The authors thank the individuals who participated in this study and the clinical staff as well as the drivers and recruiters at individual trial centers without whom the study could not have been performed; the investigators and their clinical teams for their contribution to the study and their support and care of patients.

The authors also acknowledge contributions of the Joint Steering Committee between Aeras and GSK and of the following persons within **GSK**: François Roman, Christina Caporaso, Evi De Ruymaeker, Emelia Ferreira, Florence Richard, Anne-Sophie Perreaux, Paola Pirrotta, Pramod Dhoke, Sagar Salvi, Naresh Patil, Neela Kumar, Roland Vaudry, Muriel Debois, Helen Jacob, Sophie Caterina, Mohamed Amakrane, Lieven Declerck, Marc Lievens, Hildegarde Lemaire, Stéphanie Ravault, Bruno Salaun, Nathalie Baudson, Thierry Pascal, Erik Jongert, Denis Sohy, Philippe Moris, Gerald Voss and the rest of the team; within **Aeras**: Maria Lempicki, Sebastian Gelderbloem, Maureen Lambrick, Kristin Croucher, Marisa Russell, Nathalie Cadieux, Kathryn Rutkowski, Cadwill Pillay, Sharon Sutton, Anja Van der Westhuizen, Jennie Willson, Jacqui Shea and the rest of the team; within **IQVIA**: Adele Sonnenberg, Fiona Kershaw, Gerhard Oberholzer and the rest of the team; within **Kenya Medical Research Institute**: Grace Kaguthi and the rest of the team; within the **Aurum Institute (Klerksdorp site)**: Tanya Nielson, Mathinette Taljacci, Bantubonke Ntamo, Candice Eyre, Moin Majola, Mantai Macnetha, Nollela Isreal Kunene, Weltoh Prinpili Calane, Ntshanba Tedriwd, Bonita Janse van Rensburg, Pearl Nomsa Sanyat, Audrey Lebohang Phlamini, Richard Nteleili, Moogo Fikirowo, Vernon Malay, Juanita Market, Thelma Goliani, Aneesa Lalchi, Petricine Kock, Marietjie King, Maryna Neu, Elba van Pienoburg, Mpho Macoanyane, Mnanare Tlhapi, Pontsho Motsatse, Moeti Perake, Tuelo Matotong, Boitumelo S. Kekana, Kesenogile Baepanye, Lindiwe Nhlangalele, Lawrak-Ntaahas, Palesa Masweu, Tslegafebo Saabela, Nondumiso Langa, Sehuu Matsnego, Pearl

Selepe and the rest of the team; within the **Aurum Institute (Tembisa site)**: Stacey Lawrence, Lydia Matsie Kgari, Nelly Makgotso Ratsela, Charlene Govender, Carita Marx, Phuti Mohlala, Amukelani Tshabalala, Louse Pretorius, Talifhani Saddam Mutepe, Malebo Mahapa, Nokuthula Hlophe, Zandile Mabuse, Leah Ayobami, Mpho Tshabalala, Modulakgotla Sebe, Gladys Kobane, Charlotte Checha, Nomsa Lebutse, Amogelang Ntlele, Manasa Mapendere, Nhlanhla Linkie Mokoena, Nishanee Arjun, Jane Sithole, Nomathamsanqa Mzimela, Happy Mahlangu, Rampati Sylvia Makgopa, Lindiwe Khoza, Marilize Venter, Dipono Mampho, Trevor Beattie, Morongwe Likoti, Reuben Munyai and the rest of the team; within the **Be Part Yoluntu Centre**: Eileen Fortuin, Adri Holm, Chrisna Andersen and the rest of the team; within the **Wellcome Centre for Infectious Diseases Research in Africa**: Bekekile Kwaza, Nonceba Gobe, Thembisa Ngcuka, Relebohile Tsekela, Loraine Swanepoel, Nomvula Makade, Antoneta Mashinyira, Amanda Jackson, Thelma Leopeng, Pamela van Wyhe, Nashreen Omar-Davies, Nompumelelo Ndlovu, Rene Goliath, Sandra Mukasa and the rest of the team; within the **Perinatal HIV Research Unit**: Kebafilwe Tlalang and Ziyaad Waja and the rest of the team; within the **South African Tuberculosis Vaccine Initiative**: Elma Van Rooyen, Adam Penn-Nicholson, Amary**l** Van Schalkwyk, Angelique Mouton, Ashley Veldsman, Asma Toefy, Boitumelo Mosito, Bongiwe Vazana, Charmaine Abrahams, Christal Ferus, Constance Schreuder, Cynthia Gwintsa, Danelle Van As, Denis Arendsen, Fajwa Opperman, Hadn Africa, Hennie Geldenhuys, Imogine Voight, Janelle Botes, Justin Shenje, Lebohang Makethe, Liticia Swanepoel, Lungisa Jaxa, Maigan Ratangee, Marcia Steyn, Marwou De Kock, Michele Van Rooyen, Miriam Moses, Mzwandile Erasmus, Natasja Botes, Nicole Bilek, Onke Nombida, Pamela Mangala, Portia Dlakavu, Raida Onrust, Simbarashe Mabwe, Simon Mendelsohn, Sindile Matiwane, Sonia Stryers, Susan Rossouw, Veronica Baartman and the rest of the team; within the **Setshaba Research Centre**: Khatija Ahmed, Veronique Bailey, Mduduzi Masilela, Nadia Van

Nierkerk and the rest of the team; within the **Task Applied Science**: Ida van Deventer, Erlandy Basson, Carmelita Hoffman, Priscilla Samaai, Aluta Nqipha, Elsie van der Merwe and the rest of the team; within the **Centre for Infectious Diseases Research in Zambia**: Steward Reid, Jill Morse, Bupe Sichalwe, Rhoidah Chitambi, Muyunda Siyambango, Martha Phiri, Chusi Sikanyika, Estella Kalunkumya, Moses Zimba, Inonge Mangambwa, Mpande Mukumbwa Mwenechanya, Muhau Mubiana, Ranjit Warrer, David Ojok, Kaunda Kaunda, Mwiinga Mwendalubi, Chifundo Phiri and the rest of the team; and within **Zambart**: Modupe Amofa-Sekyi, Elizabeth Biemba, Mapopa Ndhlocu, Isaac Mshanga, Chepela Ngulube and the rest of the team.

The authors also thank the IDMC members.

Writing assistance was provided by Joanne Wolter (Independent medical writer on behalf of GSK), editing and manuscript coordination services by William Zonta (XPE Pharma & Science on behalf of GSK), manuscript coordination and critical review by Stéphanie Delval (GSK) and design support by Valérie Lafontaine (Business & Decision Life Sciences on behalf of GSK).

AD is supported by a grant of the South African National Research Foundation.

RJW is supported by Wellcome (104803, 203135) Francis Crick Institute (10218).

The authors would like to honor the memory of Malibongwe Sicelo Sindelo, study nurse in Cape Town, who tragically died during the preparation of this report.

Supplementary methods

Inclusion and exclusion criteria

All participants satisfied the following criteria at study entry:

- Participants who, in the opinion of the investigator, could and would comply with the requirements of the protocol (e.g. completion of the diary cards, return for follow-up visits);
- A male or female between, and including, 18 and 50 years of age at the time of obtaining informed consent;
- Written (or thumb printed and witnessed) informed consent obtained from the participant;
- Baseline positive interferon-gamma release assay (IGRA) test result;
- Baseline negative human immunodeficiency virus (HIV) screen;
- Baseline negative clinical screening questionnaire and negative sputum sample for pulmonary tuberculosis disease;
- Healthy participants or those with chronic well controlled disease as established by medical history and clinical examination;
- Female participants of non-childbearing potential could be enrolled in the study;
  - Non-childbearing potential was defined as pre-menarche, current tubal ligation, hysterectomy, ovariectomy or post-menopause
- Female participants of childbearing potential could be enrolled in the study, if the participant:
  - had practiced adequate contraception* for 25 days prior to vaccination, and
  - had a negative pregnancy test on the day of screening and the day of first vaccination, and
  - agreed to continue adequate contraception* during the entire vaccination period and for 2 months after completion of the vaccination series.

  *Adequate contraception was defined as a contraceptive method with failure rate of less than 1% per year when used consistently and correctly and when applicable, in accordance with the product label for example:

  - abstinence from penile-vaginal intercourse, when this was their preferred and usual lifestyle,
  - oral contraceptives, either combined or progestogen alone,
  - injectable progestogen,
  - implants of etonogestrel or levonorgestrel,
  - estrogenic vaginal ring,
  - percutaneous contraceptive patches,
  - intrauterine device or intrauterine system,

- male partner sterilization prior to the female participant's entry into the study, and this male was the sole partner for that participant (the information on the male sterility could come from the site personnel's review of the subject's medical records or from interview with the participant on her medical history),
- male condom combined with a vaginal spermicide (foam, gel, film, cream or suppository),
- male condom combined with a female diaphragm, either with or without a vaginal spermicide (foam, gel, film, cream, or suppository).

Adequate contraception did not apply to subjects of child bearing potential with same sex partners, when this was their preferred and usual lifestyle.

The following criteria should be checked at the time of study entry. If any exclusion criterion applied, the participant could not be included in the study:

- Current tuberculosis (TB) disease or history of TB disease and/or treatment for TB (including isoniazid preventive therapy);
- Use of any investigational or non-registered product (drug or vaccine) other than the study vaccines within 30 days preceding the first dose of study vaccine, or planned use during the study period;
- Planned administration/administration of a vaccine not foreseen by the study protocol in the period starting 30 days before and ending 30 days after each dose of vaccine;
- History of previous administration of experimental *Mycobacterium tuberculosis* (Mtb) vaccines;
- Chronic administration (defined as more than 14 days in total) of immunosuppressants or other immune-modifying drugs within six months prior to the first vaccine dose (for corticosteroids, this meant prednisone ≥20 mg/day or equivalent). Inhaled and topical steroids were allowed;
- Any condition or illness (acute, chronic or history) or medication, which in the opinion of the investigator could interfere with the evaluation of the safety or immunogenicity of the vaccine;
- Any confirmed or suspected immunosuppressive or immunodeficient condition, based on medical history and physical examination (no laboratory testing required);.
- Planned participation or participation in another experimental protocol during the study;
- Concurrently participating in another clinical study, at any time during the study period, in which the participant had been or would be exposed to an investigational or a non-investigational product (pharmaceutical product or device);
- Administration of immunoglobulins and/or any blood products within the 3 months preceding the first dose of study vaccine or planned administration during the study period;

- History of any reaction or hypersensitivity likely to be exacerbated by any component of the vaccines;.
- History of medically confirmed autoimmune disease (e.g. type I diabetes, lupus);
- Pregnant or lactating female;
- Female planning to become pregnant or planning to discontinue contraceptive precautions during the vaccination period and/or before 2 months after completion of the vaccination series.

Screening procedures

The following procedures were conducted at the screening visit, -30 to 0 days before the first immunization:

- Recording of informed consent and check of inclusion/exclusion criteria;
- Collection of demographic data, medical history and physical examination;
- HIV testing with pre-test and post-test counselling. HIV-positive participants at screening were referred for confirmatory HIV diagnosis and management, and were not enrolled (exclusion criterion);
- Documentation of history of Bacille Calmette-Guérin (BCG) vaccination/presence of scar;
- Documentation of history of TB household contacts;
- A urine pregnancy test was performed on all female participants of childbearing potential;
- Blood sampling for QuantiFERON Gold In-Tube test;
- Sputum collection for polymerase chain reaction (PCR) testing;
- Reporting of serious adverse events (SAEs).

QuantiFERON TB Gold In-Tube assay

QuantiFERON-TB Gold In-Tube is an *in vitro* diagnostic test using peptide cocktails of ESAT-6, CFP-10 and TB7.7 proteins to stimulate cells in heparinized whole blood. These proteins are absent from all BCG strains and from most non-tuberculosis mycobacteria with the exception of *M. kansarii, M. szulgai* and *M. marinum*. The assay was performed according to the manufacturer’s instructions. Briefly, blood was collected directly into QuantiFERON-TB Gold In-Tube collection tubes including a Nil Control tube, TB antigen tube and Mitogen Control tube. The tubes were incubated for 16-24 hours at 37°C prior to harvesting plasma. Interferon (IFN)-γ concentrations in plasma were determined using the QuantiFERON-TB Gold enzyme-linked immunosorbent assay (ELISA) kit (manufacturer recommended cut-off 0.35 IU/ml).

No IGRA test was performed beyond baseline. The authors acknowledge this limitation and agree that some participants might have reverted to negative IGRA during the study. IGRA reversion has not been definitively shown to be associated with decreased risk of progression to TB disease.[1] The impact on the final analysis could be a diluted effect on

overall TB incidence associated with IGRA reversion, which would not be expected to differentially affect TB disease incidence by study arm. It is also interesting to note that the attack rate in our study was in line with the expectations and therefore, the rate of IGRA reversion may be assumed to be limited.

Composition of vaccine and placebo

0.5 ml dose of M72/AS01$_E$ contains 10μg M72 reconstituted with AS01E, a GSK proprietary Adjuvant System which contains 25 μg MPL (3-O-desacyl-4-monophosphoryl lipid A produced by GSK), 25 μg QS-21 (*Quillaja saponaria* Molina, fraction 21; licensed by GSK from Antigenics Inc., a wholly owned subsidiary of Agenus Inc., a Delaware, USA corporation), and liposomes.

The placebo consisted of sucrose 20 mg/dose in phosphate buffer pellet that was reconstituted with α-Tocopherol, squalene and Tween 80. Each dose was administered intramuscularly in the deltoid region of the arm.

Treatment allocation and randomization

Samples were randomized in blocks at GSK Biological, using MATerial EXcellence. Participants were allocated to a study group at the investigator site using an internet based randomization system. The randomization algorithm used a minimization procedure accounting for center and gender. Minimization factors had equal weight in the minimization algorithm. A random element was included in the algorithm to avoid fully deterministic allocations.

Safety monitoring plan

Planned safety review by the Safety Review Team and the Independent Data Monitoring Committee (IDMC)

A planned safety review occurred after the first 100 participants were enrolled and vaccinated. The Safety Review Team reviewed blinded safety summaries data and the IDMC reviewed the unblinded safety data, during the course of the study.

The study was conducted in a double-blind fashion and the study team and participants did not have access to unblinded individual data. Only the results per group were unblinded to the study team. In limited instances, the group can be deduced for specific participants when crossing information from different table cells, but it was considered that this does not impact the integrity of the study.

Only an external and independent statistician had access to the randomization code and generated the final analysis as well as the safety analyses for the IDMC. The IDMC members had access to the unblinded safety information 10 times during the study conduct.

Rules for vaccination

At the individual participant level,

- No second dose was to be administered in a given participant who experienced Grade 3 redness and swelling, i.e. ≥100 mm, post-dose 1, or Grade 2 or 3 respiratory adverse events post-dose 1;
- No second dose was to be administered in a given participant who developed TB disease after dose 1.

At the study level,

- An *ad hoc* IDMC review was to be called if 2 or more participants experienced related Grade 2 or higher respiratory adverse events within 90 days after any dose;
- Further enrollment and vaccination was to be suspended if within 90 days after any study vaccine dose any of the following events were observed:
    - Any related respiratory adverse event with fatal outcome,
    - Two or more participants experiencing Grade 3 related respiratory adverse events.
- Vaccination could only resume pending final GSK Vaccine Safety Monitoring Board approval.

Reporting periods for adverse events, serious adverse events and pregnancies

**Study activity**	**Screening**	**Dose 1 (V1) D 0**	**7 d post-V1 D 6**	**30 d post-V1 D 29**	**Dose 2 (V2) D 30**	**7 d post-V2 D 36**	**30 d post-V2 D 59**	**6 months post-V2 M 7**	**Study Conclusion M 36**
Reporting of solicited AEs*									
Reporting of unsolicited AEs									
Reporting of SAEs									
Reporting of SAEs related to the investigational product									
Reporting of SAEs related to study participation or concurrent GSK medication/vaccine									
Reporting of pregnancies									
Reporting of pIMDs									

V = vaccination; Post-V = post-vaccination; D = Day; M = Month; SAE = serious adverse event; pIMD = potential immune-mediated disease

* For the safety and immunogenicity subcohort only

Surveillance for pulmonary TB

Surveillance for efficacy commenced with administration of the first dose of study vaccine or placebo.

Active follow-up for safety and efficacy

In addition to scheduled study visits at the study facilities, regular contacts (every two months) with the study participants was maintained using one or more of the following methods:

- Regular interval home visits by site staff;
- Phone calls to inquire about current health status, completed by home visits if the participant could not be reached;
- 1-way short message service (SMS) reminders and/or 2-way SMS exchange.

During study visits and contacts, participants were asked if they had signs or symptoms of pulmonary TB. Based on clinical suspicion of TB, and guided by the World Health Organization (WHO) signs and symptoms algorithm [WHO, 2009], they were requested to provide 3 sputum samples, preferably taken in the morning and within 1 week, for testing for Mtb by PCR and liquid culture by Mycobacterial Growth Indicator Tube.

Passive follow-up for efficacy

Participants were informed about signs and symptoms compatible with TB at the time of informed consent and at each visit/contact. At any time during the study, if any participant suspected that he/she had signs and symptoms of TB, he/she was requested to self-report to the study center for clinical evaluation (passive follow-up). Confirmatory testing using PCR and microbiological culture was performed when indicated.

Diagnostic procedures for the detection of suspected pulmonary TB

Participants with clinical suspicion of pulmonary TB provided three respiratory sputum samples, preferably taken in the morning over a week, for testing by PCR and microbiological culture, for a total of six opportunities (tests) to provide evidence of microbiologically-positive TB disease. Participants with negative results but with continuing clinical suspicion of TB disease were treated with non-anti-tuberculosis antibiotics and followed up approximately 2 weeks later. If clinical suspicion persisted, 3 additional sputum samples, preferably taken in the morning and within a 1-week interval, were collected for additional PCR testing and microbiological culture. If TB disease could not be confirmed with PCR and/or microbiological culture, the participant could be given TB treatment based on other diagnostic tests (e.g. smear microscopy) and the judgement of the physician providing care.

Sputum samples were preferably to be collected before initiation of TB treatment. However, samples for diagnostic testing with PCR and/or microbiological culture could be collected up to 4 weeks after initiation of TB treatment. Definite Pulmonary TB cases identified from sputum samples taken after initiation of TB treatment were not included

in the primary endpoint.

All patients with confirmed TB underwent screening for diabetes (HbA1c >6.5%) and for HIV-infection. For HIV-positive patients, additional testing measured CD4+ cell counts.

Diagnostic procedures for the detection of suspected extra-pulmonary TB

Participants with clinical suspicion of extra-pulmonary TB underwent a diagnostic procedure according to local clinical practice and as a minimum, the most recent WHO recommendations in the "International Standards for TB care".[2]

PCR (Xpert MTB/RIF) for Mtb detection

The PCR (Xpert MTB/RIF) assay and the GeneXpert instrument consists of a single-use multi-chambered plastic cartridge preloaded with liquid buffers and lyophilized reagent beads necessary for sample processing, DNA extraction, and hemi-nested real-time PCR. Clinical sputum samples were treated with a NaOH and isopropanol-containing sample reagent (SR). The SR was added at a 2:1 ratio to the sputum sample or sputum pellet and incubated for 15 min at room temperature. The treated sample was transferred into the cartridge, the cartridge was loaded into the GeneXpert instrument and an automatic process completes the remaining assay steps. The assay cartridge also contained lyophilized *Bacillus globigii* spores which serve as an internal sample processing and PCR control. The spores are automatically resuspended and processed during the sample processing step, and the resulting *B. globigii* DNA is amplified during the PCR step.

The standard user interface indicates the presence or absence of *M. tuberculosis*, the presence or absence of rifampicin (RIF) resistance, and a semi-quantitative estimate of *M. tuberculosis* concentration (high, medium, low, and very low). Assays that are negative for *M. tuberculosis* and also negative for the *B. globigii* internal control are reported as invalid. The PCR assay amplifies a 192-bp segment of the *M. tuberculosis* rpoB gene in a heminested real-time PCR. The internal control heminested *B. globigii* assay is multiplexed with the *M. tuberculosis* assay. *M. tuberculosis* is detected using five overlapping molecular beacon probes (probes A to E) that are complementary to the entire 81-bp RIF resistance-determining "core" region of the wild-type rpoB gene (5, 7, 14). Mutations in the rpoB gene target inhibit hybridization of 1 or more of the rpoB-specific molecular beacons, reducing or eliminating the signal from the corresponding probes. *M. tuberculosis* is identified when at least 2 of the 5 rpoB-specific molecular beacons give a positive signal with cycle threshold (CT) values that are $\leq$38 and that differ by no more than 2 cycles. *B. globigii* DNA is detected when the single *B. globigii* molecular beacon produces a CT of <38 cycles (adapted from: Blakemore et al., 2010).[3]

Statistical analysis of vaccine efficacy

The primary analysis of efficacy used the according-to-protocol (ATP) cohort. Vaccine efficacy (VE) was estimated from a Cox proportional hazard regression model (VE=1-hazard ratio) and 90% confidence intervals (90% CIs) and Wald p-values were derived.

95% CIs were also defined as a *post-hoc* analysis. The primary analysis was unadjusted but secondary analyses evaluated the effect of potential covariates.

At final efficacy analysis, the success criterion for the primary objective was the following:

- The lower limit of the 2-sided 90% CI for VE (using a Cox regression model) against first occurrence of definite pulmonary TB disease not associated with HIV-infection, meeting the first case definition, is above 0%.

If the primary objective was met, the confirmatory secondary objective was evaluated with the following success criterion:

- The lower limit of the 2-sided 90% CI for VE (using a Cox regression model) against first occurrence of definite pulmonary TB disease not associated with HIV-infection, meeting the second case definition, is above 0%.

For all VE objectives, Kaplan-Meier survival curves were plotted and compared with the control group by means of p-values from the log rank test. VE was estimated using Cox regression.

Table S 1 Summary of available clinical data using Mtb72F or M72

	Design	Country	Population	Age (years)	Schedule	Groups		Total N	Objectives	Study conclusion
Leroux-Roels et al,[4]	Phase I/II RCT	Belgium	PPD-negative	18-50	0, 1 month		40μg M72/AS01$_B$<br>40μg M72/AS02$_A$<br>40μg Mtb72F/AS02$_A$<br>40μg M72/saline<br>AS01 alone	110	Selection of optimal antigen and adjuvant	M72/AS01$_B$ demonstrated significantly higher vaccine specific Th1 CD4+ T-cell responses than the other formulations, Polyfunctional CD4+ responses persisted until year 3
Montoya et al,[5]	Phase II RCT	The Philippines	PPD-positive ≥3-≤10mm	18-45	0, 1 month		40μg M72/AS01$_B$<br>10μg M72/AS01$_E$<br>20μg M72/AS01$_E$<br>10μg M72/AS02$_D$<br>40μg M72/Saline<br>AS01$_B$ alone	180	Dose-finding study	All three M72/AS01 formulations induced CD4+ T-cell responses of comparable magnitudes that were significantly higher than M72/AS02$_D$. 10μg M72/AS01$_E$ was selected for further development
Day et al,[6]	Phase II, open	South Africa	Mtb-infected (PPD ≥10 mm) and uninfected (PPD <10mm)	21-40	0, 1 month		10μg M72/AS01$_E$	45	IL-17 and Th1 cytokine production, investigation of T-cell populations	M72/AS01$_E$ had a clinically acceptable reactogenicity profile in Mtb-infected/uninfected adults. Immunization induced multifunctional T-cells and boosted T-cell responses primed by natural Mtb
Thacher et al, [7]	Phase I/II RCT	Switzerland	HIV+ on cART CD4+ ≥200cells/mm^3	18-50	0, 1 month		10μg M72/AS01$_E$<br>AS01 alone<br>Saline	37	Reactogenicity, safety, humoral and CMI in HIV+ individuals	M72/AS01 had a clinically acceptable reactogenicity profile and was immunogenic in HIV+ individuals.
Penn-Nicholson et al, [8]	Phase II, RCT	South Africa	Varying Mtb status (QTF)	13-17	0, 1 month		10μg M72/AS01$_E$<br>Saline	60	Reactogenicity, safety, humoral and CMI in healthy adolescents (high TB endemicity)	M72/AS01 had a clinically acceptable safety and immunogenicity profile in adolescents, supporting the move to efficacy trials.
Idoko et al,[9]	Phase II, RCT	The Gambia	BCG-vaccinated infants	2-7 months	0 or 0, 1 month	After EPI vaccines	10μg M72/AS01$_E$ (1 dose)<br>10μg M72/AS01$_E$ (2 doses)<br>Meningococcal vaccine	300	Reactogenicity, safety, in BCG-vaccinated infants, given either after completion of or in co-administration with EPI vaccines.	M72/AS01 had a clinically acceptable safety and immunogenicity profile in infants. Two doses were more immunogenic than 1. There was no evidence of interference by co-administration of M72/AS01 and EPI vaccines on immunogenicity.
						Coad-ministered with Epi vaccines	10μg M72/AS01$_E$ (1 dose)<br>10μg M72/AS01$_E$			

							(2 doses)<br>EPI only			
Kumarasamy et al, [10]	Phase II RCT	India	QFT negative or positive	18-59	0, 1 month	ART-stable CD4+ ≥250 cells/mm^3<br>ART-naïve CD4+ >350 cells/mm^3<br>HIV-ve	10μg M72/AS01$_E$<br>Saline<br>10μg M72/AS01$_E$<br>Saline<br>10μg M72/AS01$_E$<br>Saline	240	Reactogenicity, safety, humoral and CMI in Indian adults with HIV	M72/AS01 had a clinically acceptable safety and immunogenicity profile in ART-stable and ART-naive HIV-positive adults
Gillard et al [11]	Phase II, RCT	Taiwan, Estonia	TB-naïve<br><br>Treated TB<br><br>TB undergoing treatment	18-59	0, 1 month		10μg M72/AS01$_E$<br>Saline<br>10μg M72/AS01$_E$<br>Saline<br>10μg M72/AS01$_E$<br>Saline	142	Reactogenicity, safety, humoral and CMI	Recruitment terminated prematurely due to high incidence of large injection site redness/swelling reactions in M72/AS01--vaccinated adults undergoing TB treatment. No additional clinically relevant adverse events were observed (except hypersensitivity in a TB-treated-M72/AS01$_E$ recipients). Robust humoral and CMI were observed.
Van den Berg[12]	Phase II, open	Belgium	HIV-, BCG primed	18-50	0, 1 month		10μg M72/AS01$_E$	20	Profile of RNA expression, CMI, reactogenicity and safety	Days 7, 10, 14 and 17 post-vaccination were identified as suitable time points for assessing transcriptome responses to vaccination from whole blood

N = number enrolled and vaccinated, RCT = randomized controlled trial, PPD = tuberculin purified protein derivative, BCG = Bacille Calmette-Guérin, CMI = cell-mediated immunity, HIV = human immunodeficiency virus, cART = combination anti-retroviral therapy, QTF = QuantiFERON Gold In-Tube test, TB = tuberculosis.

Adjuvant System	Formulation	MPL (μg)	QS21 (μg)	Dose Volume
AS02$_A$	Oil-in-water emulsion	50	50	0.5 mL
AS02$_D$	Oil-in-water emulsion	25	25	0.5 mL
AS01$_B$	Liposomes	50	50	0.5 mL
AS01$_E$	Liposomes	25	25	0.5 mL

M72 vs Mtb72f: point mutation (serine706 to alanine706) in Mtb32A and two histidine residues added after the N-term methionine.

Table S 2 Demographic and baseline characteristics of participants (Total vaccinated cohort)

Characteristic	Category	M72/AS01E N = 1786 Value or n (%)	Placebo N = 1787 Value or n (%)
Age (years) at dose 1	Mean (SD)	28.9 (8.3)	28.9 (8.3)
	Median (range)	27.0 (18-50)	27.0 (18-50)
Gender	Female	763 (42.7)	766 (42.9)
	Male	1023 (57.3)	1021 (57.1)
Geographic Ancestry	African Heritage	1346 (75.4)	1329 (74.4)
	Other*	440 (24.6)	458 (25.6)
Country where enrolled	Kenya	268 (15)	270 (15)
	South Africa	1437 (80)	1436 (80)
	Zambia	81 (5)	81 (5)
BMI at baseline	N	1785	1783
	Mean (SD)	24.4 (7.0)	24.4 (6.3)
	Median (IQR)	22.2 (20.0-26.8)	22.4 (20.1-26.9)
	Missing	1	4
History of exposure* *	Yes	292 (16.4)	301 (16.8)
Diabetes	Yes	7 (0.4)	7 (0.4)
Chronic pulmonary condition at screening	Yes	17 (1.0)	20 (1.1)
Smoking history	Never smoked	793 (44.4)	768 (43.0)
	Past smoker	85 (4.8)	110 (6.2)
	Current smoker	831 or 832#	823 or 824#
	Current smoker some days	76 (4.3)	85 (4.8)
	Missing	1 case that remains blinded	
Previous BCG vaccination or presence of a BCG scar	Yes	1374 or 1375#	1345 or 1346#
	No	152 (8.5)	166 (9.3)
	Unknown	259 (14.5)	275 (15.4)
	Missing	1 case that remains blinded	

BCG = Bacille Calmette-Guérin, N = number of participants, n = number of participants in a given category, Value = value of the considered parameter, % = n / Number of participants with available results x 100, SD = standard deviation, BMI = body mass index, IQR = interquartile range

* 'Other' includes 1 Indian, 449 colored and 448 mixed race individuals across both groups

** Recently exposed to a household contact diagnosed and/or treated for pulmonary tuberculosis

n varies because one case remains blinded

Table S 3 Number of tests (PCR and/or culture) positive for each case under case definition 1 (According to protocol cohort for efficacy – *post-hoc* analysis)

	M72/AS01E N = 10		Placebo N = 22		Total N = 32	
**Number of tests positive**	**n**	**%**	**n**	**%**	**n**	**%**
1	5	50.0	5	22.7	10	31.3
2	1	10.0	2	9.1	3	9.4
3	0	0.0	2	9.1	2	6.3
5	1	10.0	2	9.1	3	9.4
6	3	30.0	11	50.0	14	43.8
Overall	10	100	22	100	32	100

N = number of cases according to case definition 1
n/% = number/percentage of cases in a given category
Overall = cases with at least one test positive for PCR or culture

Table S 4 P-values in the Cox regression model with group, gender, age, gender by group interaction and age by group interaction for case definition 1 (ATP cohort for efficacy)

Event type	Parameter	P-value
Definite pulmonary TB	Group	0.39
	Gender	0.68
	Age	0.13
	Group*Gender	0.31
	Group*Age	0.07

P-value = two-sided from Cox regression model; TB = tuberculosis.

Table S 5 Unsolicited events; all events and those reported by at least 1% of participants within 30 days after each dose (Total vaccinated cohort)

	M72/AS01E N = 1786				Placebo N = 1787			
			95% CI				95% CI	
Preferred Term	n	%	LL	UL	n	%	LL	UL
At least one symptom	1203.0	**67.4**	65.1	69.5	812.0	**45.4**	43.1	47.8
Headache	620.0	**34.7**	32.5	37.0	339.0	**19.0**	17.2	20.9
Injection site pain	613.0	**34.3**	32.1	36.6	74.0	**4.1**	3.3	5.2
Injection site swelling	191.0	**10.7**	9.3	12.2	7.0	**0.4**	0.2	0.8
Pyrexia	122.0	**6.8**	5.7	8.1	22.0	**1.2**	0.8	1.9
Dizziness	114.0	**6.4**	5.3	7.6	90.0	**5.0**	4.1	6.2
Fatigue	113.0	**6.3**	5.2	7.6	51.0	**2.9**	2.1	3.7
Myalgia	83.0	**4.6**	3.7	5.7	24.0	**1.3**	0.9	2.0
Chills	72.0	**4.0**	3.2	5.1	8.0	**0.4**	0.2	0.9
Back pain	68.0	**3.8**	3.0	4.8	32.0	**1.8**	1.2	2.5
Upper respiratory tract infection	66.0	**3.7**	2.9	4.7	72.0	**4.0**	3.2	5.0
Injection site erythema	65.0	**3.6**	2.8	4.6	1.0	**0.1**	0.0	0.3
Influenza	62.0	**3.5**	2.7	4.4	51.0	**2.9**	2.1	3.7
Pain	61.0	**3.4**	2.6	4.4	10.0	**0.6**	0.3	1.0
Abdominal pain	58.0	**3.2**	2.5	4.2	47.0	**2.6**	1.9	3.5
Malaria	52.0	**2.9**	2.2	3.8	52.0	**2.9**	2.2	3.8
Nausea	47.0	**2.6**	1.9	3.5	24.0	**1.3**	0.9	2.0
Diarrhea	45.0	**2.5**	1.8	3.4	31.0	**1.7**	1.2	2.5
Malaise	35.0	**2.0**	1.4	2.7	5.0	**0.3**	0.1	0.7
Vomiting	34.0	**1.9**	1.3	2.7	15.0	**0.8**	0.5	1.4
Feeling hot	33.0	**1.8**	1.3	2.6	15.0	**0.8**	0.5	1.4
Chest pain	32.0	**1.8**	1.2	2.5	20.0	**1.1**	0.7	1.7
Decreased appetite	31.0	**1.7**	1.2	2.5	12.0	**0.7**	0.3	1.2
Cough	31.0	**1.7**	1.2	2.5	39.0	**2.2**	1.6	3.0
Arthralgia	30.0	**1.7**	1.1	2.4	20.0	**1.1**	0.7	1.7
Injection site pruritus	27.0	**1.5**	1.0	2.2	5.0	**0.3**	0.1	0.7
Pain in extremity	26.0	**1.5**	1.0	2.1	17.0	**1.0**	0.6	1.5
Abdominal pain upper	23.0	**1.3**	0.8	1.9	29.0	**1.6**	1.1	2.3
Rhinitis	23.0	**1.3**	0.8	1.9	29.0	**1.6**	1.1	2.3
Asthenia	18.0	**1.0**	0.6	1.6	7.0	**0.4**	0.2	0.8
Toothache	16.0	**0.9**	0.5	1.5	21.0	**1.2**	0.7	1.8
Rash	16.0	**0.9**	0.5	1.5	17.0	**1.0**	0.6	1.5

At least one symptom = at least one symptom experienced (regardless of the MedDRA Preferred Term)
N = number of participants with at least one administered dose
n/% = number/percentage of participants reporting the symptom at least once
95% CI = exact 95% confidence interval; LL = lower limit, UL = upper limit

Table S 6 Local and general solicited symptoms, all and grade 3, occurring until day 7 post-vaccination (sub-cohort, Total vaccinated cohort)

		M72/AS01E				Placebo			
				95% CI				95% CI	
**Symptom**	**Intensity**	n	%	LL	UL	n	%	LL	UL
		**Dose 1**							
		**N=148**				**N=151**			
Pain	All	110	**74.3**	66.5	81.1	48	**31.8**	24.5	39.9
	Grade 3	19	**12.8**	7.9	19.3	4	**2.6**	0.7	6.6
Redness (mm)	All	12	**8.1**	4.3	13.7	0	**0.0**	0.0	2.4
	>50	2	**1.4**	0.2	4.8	0	**0.0**	0.0	2.4
	>100	1	**0.7**	0.0	3.7	0	**0.0**	0.0	2.4
Swelling (mm)	All	25	**16.9**	11.2	23.9	1	**0.7**	0.0	3.6
	>50	9	**6.1**	2.8	11.2	0	**0.0**	0.0	2.4
	>100	1	**0.7**	0.0	3.7	0	**0.0**	0.0	2.4
Fatigue	All	90	**60.8**	52.5	68.7	63	**41.7**	33.8	50.0
	Grade 3	18	**12.2**	7.4	18.5	11	**7.3**	3.7	12.7
Headache	All	80	**54.1**	45.7	62.3	60	**39.7**	31.9	48.0
	Grade 3	16	**10.8**	6.3	17.0	9	**6.0**	2.8	11.0
Malaise	All	67	**45.3**	37.1	53.7	29	**19.2**	13.3	26.4
	Grade 3	14	**9.5**	5.3	15.4	9	**6.0**	2.8	11.0
Myalgia	All	74	**50.0**	41.7	58.3	34	**22.5**	16.1	30.0
	Grade 3	16	**10.8**	6.3	17.0	5	**3.3**	1.1	7.6
Respiratory symptoms	All	22	**14.9**	9.6	21.6	10	**6.6**	3.2	11.8
	Grade 3	0	**0.0**	0.0	2.5	2	**1.3**	0.2	4.7
Temperature/(Axillary)	All (≥37.5°C)	33	**22.3**	15.9	29.9	16	**10.6**	6.2	16.6
	>38.0°C	10	**6.8**	3.3	12.1	7	**4.6**	1.9	9.3
	>39.5°C	2	**1.4**	0.2	4.8	2	**1.3**	0.2	4.7
		**Dose 2**							
		**N=130**				**N=141**			
Pain	All	86	**66.2**	57.3	74.2	22	**15.6**	10.0	22.7
	Grade 3	29	**22.3**	15.5	30.4	1	**0.7**	0.0	3.9
Redness (mm)	All	5	**3.8**	1.3	8.7	2	**1.4**	0.2	5.0
	>50	1	**0.8**	0.0	4.2	0	**0.0**	0.0	2.6
	>100	0	**0.0**	0.0	2.8	0	**0.0**	0.0	2.6
Swelling (mm)	All	13	**10.0**	5.4	16.5	2	**1.4**	0.2	5.0
	>50	6	**4.6**	1.7	9.8	0	**0.0**	0.0	2.6
	>100	1	**0.8**	0.0	4.2	0	**0.0**	0.0	2.6
Fatigue	All	69	**53.1**	44.1	61.9	31	**22.0**	15.5	29.7
	Grade 3	15	**11.5**	6.6	18.3	3	**2.1**	0.4	6.1
Headache	All	74	**56.9**	48.0	65.6	39	**27.7**	20.5	35.8
	Grade 3	24	**18.5**	12.2	26.2	4	**2.8**	0.8	7.1
Malaise	All	56	**43.1**	34.4	52.0	21	**14.9**	9.5	21.9
	Grade 3	19	**14.6**	9.0	21.9	3	**2.1**	0.4	6.1
Myalgia	All	55	**42.3**	33.7	51.3	21	**14.9**	9.5	21.9
	Grade 3	18	**13.8**	8.4	21.0	0	**0.0**	0.0	2.6
Respiratory symptoms	All	14	**10.8**	6.0	17.4	5	**3.5**	1.2	8.1
	Grade 3	2	**1.5**	0.2	5.4	0	**0.0**	0.0	2.6
Temperature/(Axillary)	All (≥37.5°C)	37	**28.5**	20.9	37.0	10	**7.1**	3.5	12.7
	>38.0°C	18	**13.8**	8.4	21.0	4	**2.8**	0.8	7.1
	>39.5°C	4	**3.1**	0.8	7.7	0	**0.0**	0.0	2.6
		**Overall per participant**							
		**N=148**				**N=151**			
Pain	All	121	**81.8**	74.6	87.6	52	**34.4**	26.9	42.6

	Grade 3	36	**24.3**	17.7	32.1	5	**3.3**	1.1	7.6
Redness (mm)	All	16	**10.8**	6.3	17.0	2	**1.3**	0.2	4.7
	>50	3	**2.0**	0.4	5.8	0	**0.0**	0.0	2.4
	>100	1	**0.7**	0.0	3.7	0	**0.0**	0.0	2.4
Swelling (mm)	All	34	**23.0**	16.5	30.6	3	**2.0**	0.4	5.7
	>50	13	**8.8**	4.8	14.6	0	**0.0**	0.0	2.4
	>100	2	**1.4**	0.2	4.8	0	**0.0**	0.0	2.4
Fatigue	All	102	**68.9**	60.8	76.3	71	**47.0**	38.9	55.3
	Grade 3	27	**18.2**	12.4	25.4	13	**8.6**	4.7	14.3
Headache	All	102	**68.9**	60.8	76.3	70	**46.4**	38.2	54.6
	Grade 3	34	**23.0**	16.5	30.6	12	**7.9**	4.2	13.5
Malaise	All	86	**58.1**	49.7	66.2	40	**26.5**	19.6	34.3
	Grade 3	27	**18.2**	12.4	25.4	11	**7.3**	3.7	12.7
Myalgia	All	88	**59.5**	51.1	67.4	45	**29.8**	22.6	37.8
	Grade 3	30	**20.3**	14.1	27.7	5	**3.3**	1.1	7.6
Respiratory symptoms	All	29	**19.6**	13.5	26.9	13	**8.6**	4.7	14.3
	Grade 3	2	**1.4**	0.2	4.8	2	**1.3**	0.2	4.7
Temperature/(Axillary)	All (≥37.5°C)	58	**39.2**	31.3	47.5	23	**15.2**	9.9	22.0
	>38.0°C	28	**18.9**	13.0	26.2	10	**6.6**	3.2	11.8
	>39.5°C	6	**4.1**	1.5	8.6	2	**1.3**	0.2	4.7

For each dose and overall/participant:
N = number of participants with at least one documented dose
n/% = number/percentage of participants reporting the symptom at least once
95% CI = exact 95% confidence interval; LL = lower limit, UL = upper limit
Grade 3 or 'severe' symptoms were defined as pain that was significant at rest and prevented normal everyday activities; redness and swelling ≥100 mm, temperature >39.5°C, and as preventing normal activity for all other symptoms.

Table S 7 Concordance of PCR and microbiological culture tests for sputum samples from all suspected pulmonary tuberculosis cases (Total vaccinated cohort for Efficacy)

		Microbiological Culture Result		
		**Positive**	**Negative**	**Total**
**Xpert MTB/RIF Result**	Positive	True positive 81	False positive 12	Positive predictive value = 0.87
	Negative	False negative 20	True negative 1409	Negative predictive value = 0.99
	**Total**	Sensitivity = 0.80	Specificity = 0.99	

.
Culture is used as standard method in this comparison

Figure S 1 **Percentage of participants outside the normal ranges and at the different grades of severity for hematology, biochemistry (Total vaccinated cohort in the immunogenicity subset, grading 1-4 according to FDA standards)**

Some results remain blinded. For these cases the result has been allocated to both study groups (indicated as *), thus showing the highest possible percentage in each group

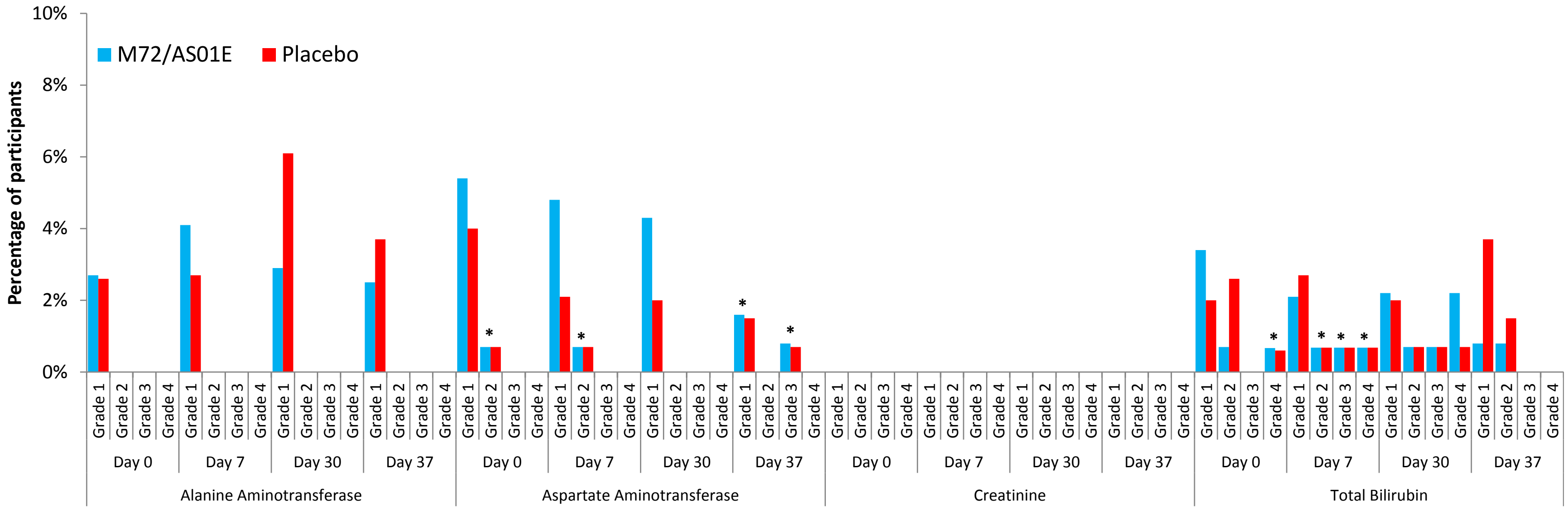

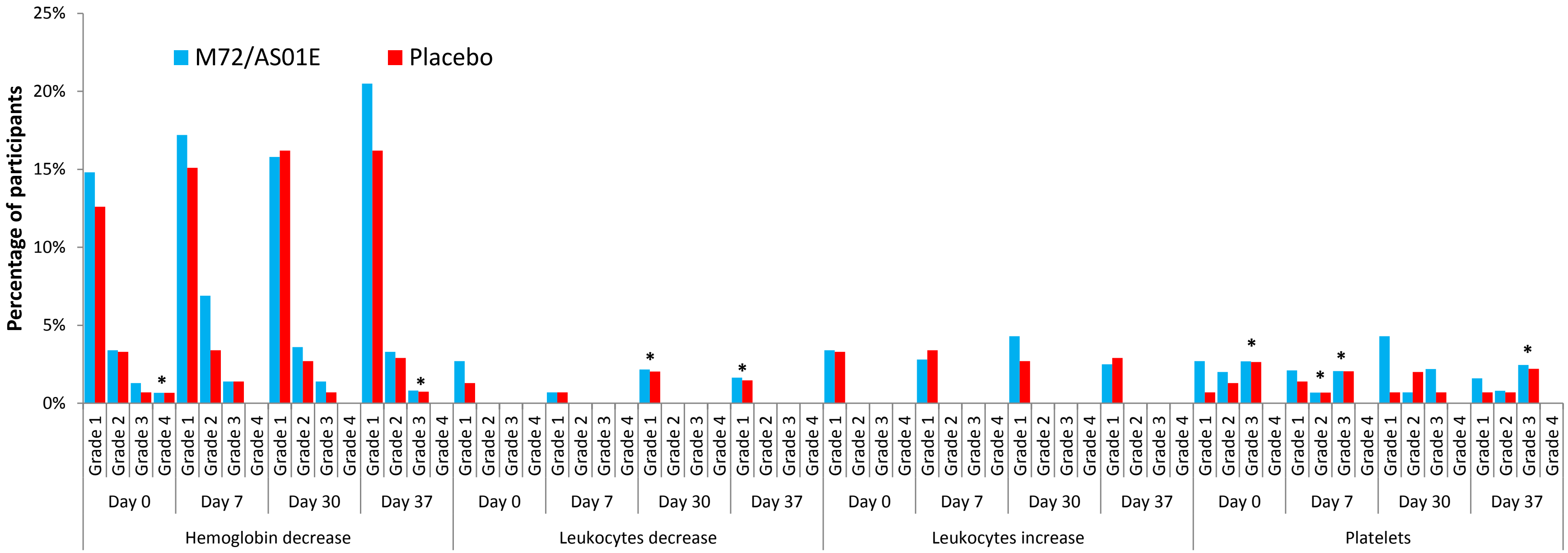
25%
20%
15%
10%
5%
0%
Percentage of participants
M72/AS01E
Placebo
Grade 1
Grade 2
Grade 3
Grade 4
Day 0
Day 7
Day 30
Day 37
Hemoglobin decrease
Leukocytes decrease
Leukocytes increase
Platelets
*

Figure S 2 Immunogenicity before and one month and 12 months post-dose 2 (ATP immunogenicity sub-cohort)

GMC = geometric mean antibody concentration. All participants with a value below the assay cut-off are assigned a value of half the cutoff for the purposes of GMC calculation.

All (100%) M72/AS01$_E$ recipients were seropositive for anti-M72 IgG antibodies one month post-dose 2 compared to 9.1% prior to vaccination. Compared to pre-vaccination levels, anti-M72 antibody GMCs increased by 340-fold after dose 2, and remained 26-fold higher than pre-vaccination levels at month 12. No change in the antibody response was observed in placebo recipients.

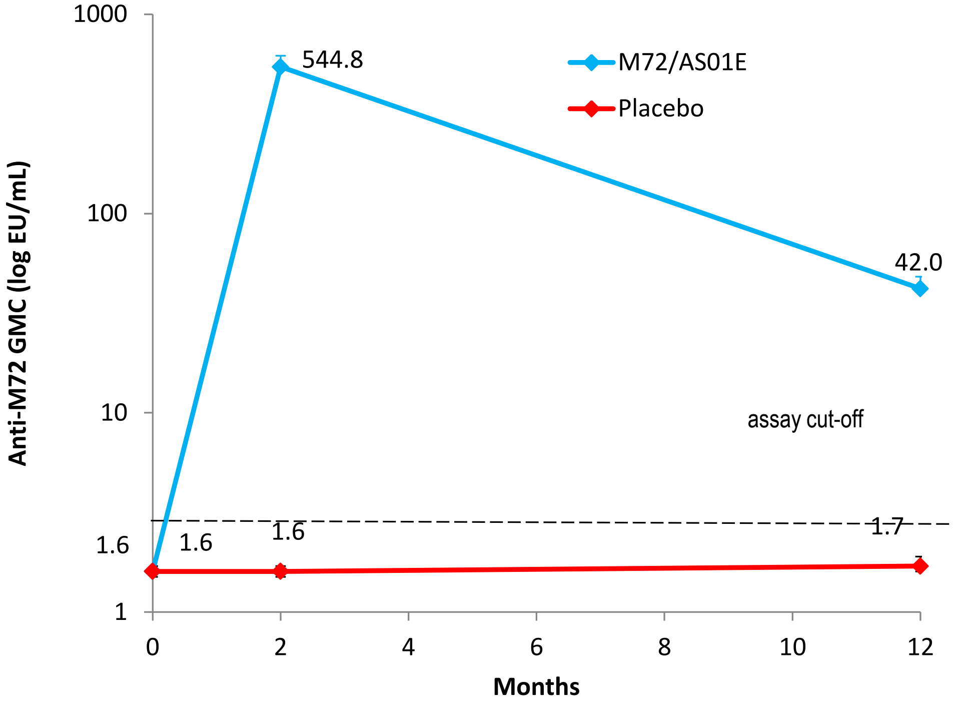

Figure S 3: “Focus on the Patient” section

Focus on the Patient

What is the context?

An estimated one quarter of the world’s population is already latently infected with *Mycobacterium tuberculosis* (Mtb), and represents a very large reservoir of future active and infectious tuberculosis (TB) cases.

The WHO goal to reduce the global burden of TB will not be achievable without a new vaccine.

An adjuvanted TB candidate vaccine M72/AS01$_E$, is currently being investigated with the primary target being adolescents and adults in TB endemic regions since these age groups are the major transmitters of Mtb.

What is new?

In this proof-of-concept study, M72/AS01$_E$ reduced by half the risk of developing tuberculosis disease in adults already infected with Mtb.

This is the first successful efficacy trial of a subunit TB vaccine against TB disease.

What is the impact?

This study provides the strongest evidence to date that a vaccine can prevent TB in already Mtb-infected adults, and this may be a valuable approach to helping to control the global TB epidemic.

This trial may also provide a unique opportunity to better understand the mechanisms by which a vaccine confers protection against TB.

Supplementary references

1. Andrews JR, Hatherill M, Mahomed H, et al. The dynamics of QuantiFERON-TB gold in-tube conversion and reversion in a cohort of South African adolescents. Am J Respir Crit Care Med 2015;191:584-91.
2. World Health Organisation. International Standards for Tuberculosis Care: Diagnosis, Treatment, Public Health. 2006. Available from http://www.who.int/tb/publications/2006/istc_report.pdf), accessed 02 April 2012.
3. Blakemore R, Story E, Helb D, et al. Evaluation of the analytical performance of the Xpert MTB/RIF assay. J Clin Microbiol 2010;48:2495-501.
4. Leroux-Roels I, Forgus S, De Boever F, et al. Improved $CD4^+$ T cell responses to *Mycobacterium tuberculosis* in PPD-negative adults by M72/AS01 as compared to the M72/AS02 and Mtb72F/AS02 tuberculosis candidate vaccine formulations: a randomized trial. Vaccine 2013;31:2196-206.
5. Montoya J, Solon JA, Cunanan SR, et al. A randomized, controlled dose-finding Phase II study of the M72/AS01 candidate tuberculosis vaccine in healthy PPD-positive adults. Journal of Clinical Immunology 2013;33:1360-75.
6. Day CL, Tameris M, Mansoor N, et al. Induction and regulation of T-cell immunity by the novel tuberculosis vaccine M72/AS01 in South African adults. Am J Respir Crit Care Med 2013;188:492-502.
7. Thacher EG, Cavassini M, Audran R, et al. Safety and immunogenicity of the M72/AS01 candidate tuberculosis vaccine in HIV-infected adults on combination antiretroviral therapy: a phase I/II, randomized trial. AIDS 2014;28:1769-81.
8. Penn-Nicholson A, Geldenhuys H, Burny W, et al. Safety and immunogenicity of candidate vaccine M72/AS01E in adolescents in a TB endemic setting. Vaccine 2015;33:4025-34.
9. Idoko OT, Owolabi OA, Owiafe PK, et al. Safety and immunogenicity of the M72/AS01 candidate tuberculosis vaccine when given as a booster to BCG in Gambian infants: an open-label randomized controlled trial. Tuberculosis (Edinb) 2014;94:564-78.
10. Kumarasamy N, Poongulali S, Bollaerts A, et al. A Randomized, Controlled Safety, and Immunogenicity Trial of the M72/AS01 Candidate Tuberculosis Vaccine in HIV-Positive Indian Adults. Medicine (Baltimore) 2016;95:e2459.
11. Gillard P, Yang PC, Danilovits M, et al. Safety and immunogenicity of the M72/AS01E candidate tuberculosis vaccine in adults with tuberculosis: A phase II randomised study. Tuberculosis (Edinb) 2016;100:118-27.
12. van den Berg RA, De Mot L, Leroux-Roels G, et al. Adjuvant-Associated Peripheral Blood mRNA Profiles and Kinetics Induced by the Adjuvanted Recombinant Protein Candidate Tuberculosis Vaccine M72/AS01 in Bacillus Calmette-Guerin-Vaccinated Adults. Frontiers in immunology 2018;9:564.
